# Effects of a Workplace Intervention Targeting Psychosocial Risk Factors on Safety and Health Outcomes

**DOI:** 10.1155/2015/836967

**Published:** 2015-10-18

**Authors:** Leslie B. Hammer, Donald M. Truxillo, Todd Bodner, Jennifer Rineer, Amy C. Pytlovany, Amy Richman

**Affiliations:** ^1^Portland State University, P.O. Box 751, Portland, OR 97207-0751, USA; ^2^Work Family Directions, 303 Wyman Street, Suite 300, Office No. 380, Waltham, MA 02451, USA

## Abstract

The goal of this study was to test the effectiveness of a workplace intervention targeting work-life stress and safety-related psychosocial risk factors on health and safety outcomes. Data were collected over time using a randomized control trial design with 264 construction workers employed in an urban municipal department. The intervention involved family- and safety-supportive supervisor behavior training (computer-based), followed by two weeks of behavior tracking and a four-hour, facilitated team effectiveness session including supervisors and employees. A significant positive intervention effect was found for an objective measure of blood pressure at the 12-month follow-up. However, no significant intervention results were found for self-reported general health, safety participation, or safety compliance. These findings suggest that an intervention focused on supervisor support training and a team effectiveness process for planning and problem solving should be further refined and utilized in order to improve employee health with additional research on the beneficial effects on worker safety.

## 1. Introduction

Work-life stress and poor safety communication are psychosocial risk factors that have been identified to contribute to decreased health and safety of workers. Workplace interventions focused on increasing supervisor support for work-life balance and safety communication have proven to be effective for reducing such risks (e.g., [1, 2]). Furthermore, it has been argued that strategies that take a Total Worker Health (TWH) approach may be the most effective way to improve the health and safety of workers, addressing both health promotion and health protection in an integrative fashion. The National Institute for Occupational Safety and Health (NIOSH) defines TWH as “a strategy integrating occupational safety and health protection with health promotion to prevent worker injury and illness and to advance health and well-being” [3]. However, published studies on the effectiveness of TWH programs remain scant [4].

The present study addresses this gap by assessing a TWH intervention, the Safety and Health Improvement Program (SHIP), designed to address work-family stress and safety risk factors. We examine the effectiveness of SHIP using a sample of construction workers, a sector and demographic group that the National Occupational Research Agenda [5] has targeted as understudied. Although there is recognition that managing work and family roles is challenging for workers and their families and that these challenges lead to diminished worker health and safety (e.g., [6, 7]), few workplace interventions that specifically address work-life stress and safety communication have been developed based on theory and they have not been systematically tested using scientifically sound experimental designs (for exceptions, see [2, 8–10]). Further, while the effects of supervisor behaviors, team, and organizational climate have been shown to affect a number of safety outcomes [11], relatively few studies have examined actual safety interventions other than Zohar and Luria [2], and no published intervention studies have taken a TWH approach that integrates both work-life stress and safety risk factors. Therefore, the present study addresses this gap in the research by examining the effects of a TWH intervention that addresses work-life stress and safety on worker health, well-being, and safety outcomes using a randomized control design in a sample of construction workers.

The need for psychosocial workplace interventions to promote and protect construction worker health and safety was recently illustrated by Bodner et al. [12] where work-family stress and conflict were significantly and positively correlated with diastolic blood pressure, body mass index, and pain reports. Likewise, work-family stress and conflict were significantly related to missing work in the past six months due to an injury. Although these relationships reported were correlational and, at one point in time, they suggest that construction workers are a vulnerable population and that interventions that reduce psychosocial risk factors such as targeting support for work-life balance and support for safety should be examined for their beneficial effects on worker health and safety.

## 2. Evaluation of Workplace Interventions: Process Evaluations and Effects Evaluation

Nielsen et al. [13] discussed the importance of process evaluation to intervention research and provided a discussion of the issues to consider when combining process evaluation and effect evaluation data (i.e., evaluation of how an intervention works versus an evaluation of what intervention works). It has been rightly argued that the context within which intervention takes place needs to be considered to fully understand the effects of an intervention. This includes the assessment of intervention fidelity as well as the complex contextual environment that changes from group to group and may never be able to be controlled in a group randomized design, for example. Using mixed methods designs is one way of helping to triangulate the process evaluation and effect evaluation data.

Furthermore, Biron et al.'s [14] work identified several factors that potentially contribute to limited intervention effects. They suggest that process evaluations should be conducted during intervention rollouts to better understand the role of (1) organizational contextual influences such as readiness for change; (2) the possibility that the introduction of an intervention is perceived as a job demand, leading to decreased well-being rather than to the expected beneficial effects the researchers had hoped for; (3) low ownership by stakeholders; and (4) characteristics of the intervention such as scope, approach, and target which may exceed existing organizational resources.

This idea extends to the four levels of training criteria discussed by Kirkpatrick [15], running from those most easily affected by training (reactions) to critical outcomes (results) that are the ultimate goals of training programs. Specifically,* reactions* refer to training participants' affective response to the training;* learning* refers to improvement in knowledge and skills after training;* behavior* refers to changes in behavior such as transfer of learned knowledge and skills back to the job; the ultimate training criterion,* results*, refers to changes in important organizational outcomes, such as organizational productivity or, in TWH terms, participant health. Using this framework, in the present study, our focus was on the three highest levels, specifically, changes in learning due to the computer-based training of supervisors; behavior in terms of transfer of knowledge and skills back to the job through behavior tracking and the team effectiveness process; and results in terms of improvements in employee health and safety.

Thus, using a combination of qualitative and quantitative methods, we developed an evaluation of SHIP that included both process and effect assessments. We assessed follow-up and uptake of the intervention within workgroups at 30, 60, and 90 days after intervention through inspection of notes generated from check-in meetings. Furthermore, consistent with training research, we argue that it is critical for training to include a design that fosters motivation to transfer the training content to the job (e.g., [16, 17]). As part of our training design, we incorporated behavioral self-monitoring using iPods that tracked behaviors learned during the training. This has been a proven method to enhance transfer of training in prior research (see [18]). Behavioral self-monitoring is a technique in which individuals repeatedly observe, evaluate, and record aspects of their own behavior.

## 3. Work-Family Psychosocial Risk Factors and Health

Work-family/life stressors are rising for nearly every demographic and occupational group in the U.S. [19]. There is growing recognition that work-family stressors have risen for workers and their families across the nation, leading to decreased health of workers and their family members [20, 21]. In addition, escalating time pressures and work-family conflict have negative business consequences such as reduced worker productivity and turnover [22–24] and negative long-term consequences for the economic health of organizations and, ultimately, society. Effects of psychosocial factors such as work-life stress on the health of workers have been documented, as have the effects of such stress and conflict on health behaviors, precursors to chronic health outcomes [7]. Furthermore, work-life stress has been shown to be related to worker safety outcomes [6, 25, 26]. Thus, work-life stress is a psychosocial risk factor and is identified as an occupational hazard by Hammer and Sauter [7]. In turn, supervisor support for work and family is related to reductions in work-life stress [8, 9].

Work-family conflict and stress are linked to general mental and physical health outcomes [27–32]; more chronic physical symptoms; and higher levels of dysphoria, psychological distress, and sickness absence [33, 34]. Other studies suggest that, over time, the effects of work-family stress result in negative health outcomes among objectively measured indicators such as high blood pressure [35, 36] and other mental and physical health problems [37–39].

In the present study, a primary outcome of interest is blood pressure, a known risk factor for cardiovascular disease [40]. Although many factors can contribute to high blood pressure, the impact of work-related psychosocial risk factors, such as job strain, on blood pressure and cardiovascular disease is established [41–44]. However, there are also workplace factors that can help buffer these effects. One mechanism shown to help decrease the relationship between job strain and cardiovascular disease and its risk factors is social support [45, 46]. For example, research has demonstrated that higher levels of supervisor support are related to lower levels of blood pressure and improved sleep [47, 48].

Building upon this, there are multiple types of social support that may influence worker health: spousal support, coworker support, and supervisor support. For the purposes of this discussion, we will focus on the supervisor support construct developed by Hammer and colleagues [49] called family-supportive supervisory behaviors (FSSB). This is a form of social support focused on supervisors providing support specifically to workers to assist with the integration of work and family, thereby reducing work-life stress and related strain outcomes. A central component of the intervention in the present study is a focus on reducing work-life stress through training supervisors to focus on FSSB.

This emphasis on training the supervisor is based on our earlier work that conceptualizes the supervisor as the linking pin or the key critical organizational level that impacts health and well-being of workers. We based the development of the intervention, supervisor supportive training, on the FSSB concept. FSSB is made up of 4 types of support based on the work of Hammer and colleagues [49], and this is represented in the training intervention that contained training on the FSSB dimensions of emotional support, instrumental support, work-family role modeling, and work-family creative management. In addition, the intervention involved a team-based work design change process that directly involved the workgroup employees as described in the Method section of the paper. The intervention consisted of two change approaches, a top-down approach that focuses on the supervisors (computer-based training) and a bottom-up approach that focuses on the workgroup members (team effectiveness process). An advantage of this approach is that it increases the odds of creating positive change in organizations; a disadvantage of this approach is that we cannot attribute any intervention effects to either of the two components. Thus, this intervention was designed to have both a top-down (i.e., supervisor-based) and bottom-up (i.e., employee-based) components. This multilevel intervention design approach is expected to have a stronger effect on intervention outcomes compared to one level or another.

While few work-family interventions have been developed based on theory and research and evaluated using scientifically sound designs that integrate measurement of the intervention's effects on safety and health outcomes [50, 51], there have been increasing employer interest in and experimentation with creating supportive work-family workplaces and flexible work arrangements, schedules, and other work-life and “family friendly” policies [42–44, 52–54]. However, most employers find it challenging to know how to effectively implement these new ways of working [55] and, specifically, which interventions are most effective. Moreover, rigorous evaluations of work-family programs and policies that involve longitudinal data and appropriate comparison groups are virtually nonexistent. Identifying and testing such workplace interventions to reduce work-family stress and conflict is an important public health issue, given the significant effects of high work-family conflict on the health and well-being of workers and their families (e.g., [1, 33]). Further, increasing organizational support for work and family through supportive managers and workplace cultures and through increasing employees' involvement in developing strategies for eliminating low-value work may have significant implications for the health and well-being of workers.

## 4. Safety-Related Psychosocial Risk Factors and Safety Outcomes

Similarly, little is known about workplace interventions that are implemented to improve safety communication and climate, other than the work of Zohar and colleagues on supervisor safety communication strategies (e.g., [2, 10, 56]). While there is a substantial literature regarding the consistent effects of safety climate and leadership on safety behaviors and attitudes (e.g., [11, 57–59]), few studies have addressing safety climate training and interventions, and none have combined work-life stress reduction with improvements in supervisor safety communication.

Safety is a critical outcome, especially in high-risk occupations such as construction. Safety outcomes are determined by more than workplace environmental and individual behavior factors. As research is beginning to show, psychosocial workplace factors such as work-life stress and conflict, as well as poor safety communication and climate, also affect safety outcomes [2, 6, 25, 26].

At least three critical meta-analyses have demonstrated the link between safety climate (shared employee perceptions of the safety environment at the organizational and group level, as well as individual perceptions of climate) and safety outcomes [11, 57, 58]. It has further been argued that safety climate is determined by supervisory practices, communications, and behaviors (e.g., [59]). For example, Zohar [60] found that transformational leadership focused on follower welfare was important to safety outcomes. Accordingly, Zohar [61] used an intervention focused on increasing safety interactions in teams so that safety would be seen as having as much priority as production. Feedback from the next level of supervisors was also included. The intervention increased safety-related interactions, improved safety culture, and decreased minor accidents. Zohar and Luria [2] also found that monitoring and providing feedback to supervisors about safety-related interactions improved safety behavior and safety climate. Therefore, the development of an intervention that targets supervisory behaviors is likely to improve safety climate, which has been shown meta-analytically to affect safety behaviors by increasing worker safety motivation and safety knowledge [11].

Griffin and Neal [62] break down the construct of safety behaviors (performance) into two subdimensions: safety compliance and safety participation. Safety compliance refers to engaging in core safety behaviors that are central to the maintenance of a safe working environment, such as wearing safety goggles. Safety participation consists of more contextual behaviors that contribute to an overall environment of organizational safety, such as helping coworkers or volunteering for safety-related activities. In the present study, safety will be operationalized using these two dimensions [63].

## 5. The Present Study

The present study examines an integrated work-family and safety support intervention, SHIP, over time within a vulnerable worker population, construction workers. Past research has used white collar and retail samples to assess the effects of family support from supervisors. In contrast, in this study we advance the literature by examining the effects of this intervention on blood pressure, self-reported health, safety participation behavior, and safety compliance behavior in a construction worker sample. Construction work is a safety-sensitive occupation and, as such, we focused on work-life conflict and safety communication training. Construction workers have more rigid schedules than professional-level workers who may experience high levels of work-life stress but who may have more schedule flexibility to manage such stress. Furthermore, construction work is a male-dominated profession and the expectation for hiding or limiting one's work-life stress may be an additional subtle occupational pressure. Furthermore, construction is a highly dangerous job with many of the injuries in the US occurring in construction. Thus, this is an appropriate sample for examining work-life and safety hazards.

Furthermore, this research is part of a larger research program on supervisor support interventions and specifically family-supportive supervisor behaviors (FSSB) conducted by Hammer and colleagues [8, 49]. We have extended this training paradigm to also include supervisor support for safety and thus, with training focused on work-life conflict and safety, we chose theoretically relevant outcome variables. Furthermore, some of our prior research has demonstrated that work-life conflict, specifically family-to-work conflict, was related to safety participation outcomes.

Construction workers complete physically demanding tasks on the job, but few studies have examined the negative health and safety effects related to psychosocial aspects of their work (for an exception, see [12]). To address this from a TWH perspective, SHIP was developed to improve worker health and safety through reductions in occupational stress via improved supervisor support and team effectiveness. These intervention components are grounded in theory from multiple disciplines and are partially supported by findings from pilot/feasibility studies conducted by the Work, Family, and Health Network (http://www.workfamilyhealthnetwork.com/), as well as by the work of Zohar and colleagues.

The FSSB training intervention developed by Hammer and colleagues [8] as well as the supervisor-based safety training intervention developed by Zohar [61] was used as the basis for the development of the integrated training approach, SHIP, in the present study. Our behavior tracking strategy was informed by Olson and Winchester's [18] study demonstrating the effectiveness of this method for improving training transfer. In addition, we drew on the suggestion of Zohar and Luria [59], as well as that of Kelly and colleagues [9], to integrate work teams into the change process. Thus, we used the team effectiveness process (TEP) developed by Work Family Directions (WFD Consulting), a consulting firm that specializes in work-family integration practices within organizations. The TEP process has been used in a variety of industries with employees in many types of jobs including hospitality, financial services, technology, engineering, manufacturing, call centers, and sales.

In sum, the purpose of this study is to examine the effects of an integrated, theory-based work-life and safety TWH intervention. SHIP targets the psychosocial work environment through training supervisors and work team members to decrease the psychosocial risk factors of work-life stress and poor safety communication, with expected effects on the health (e.g., blood pressure), well-being, and safety of workers. Specifically, the components of SHIP are (1) family-supportive supervisor behavior (FSSB) and supervisor-based safety (SBS) training, and (2) posttraining tracking of learned behaviors, (3) team effectiveness process for planning and problem solving (TEP), and (4) monthly posttraining check-ins to revisit goals and assess progress. Overall, SHIP was designed to increase work-life support and improve safety and health. We examined the effectiveness of SHIP using a randomized control design with construction workers employed in a municipal city utility department. In the present study, we test whether the SHIP intervention to increase workplace support and decrease stress improves employees' safety, health, and well-being. We hypothesize that SHIP will lead to improvements in worker safety (H1) and health (H2) over time.

## 6. Method

### 6.1. Participants and Design

**Figure 1:**
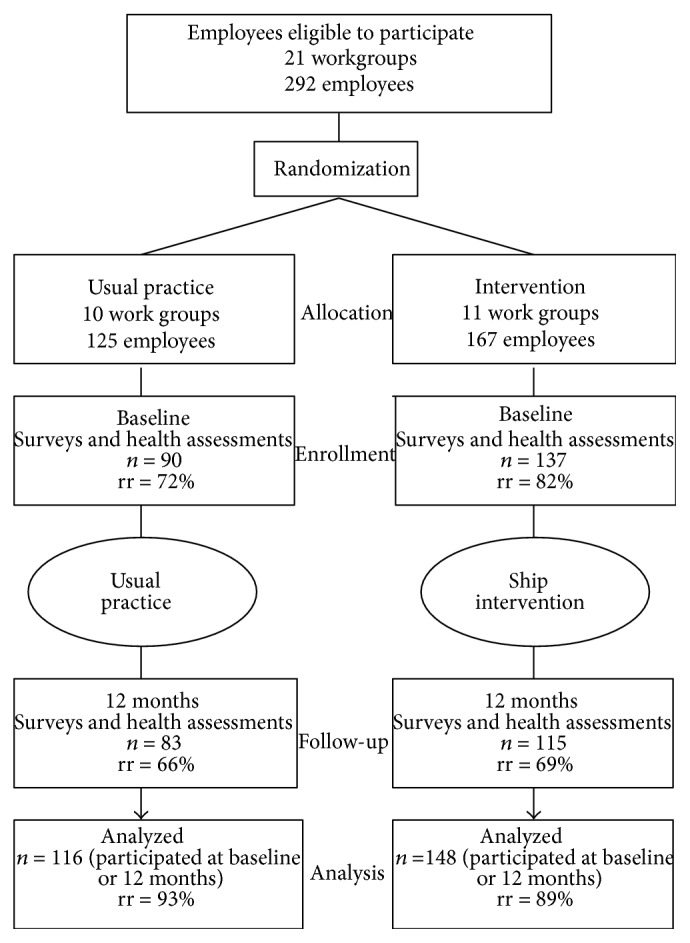
Consort diagram for SHIP randomized control trial. Rr: retention rate.

Participants were construction and utility workers in a municipal public works department. Job roles of participants included, but were not limited to, utility worker, electrician, plumber, carpenter, heavy equipment operator, and sidewalk repair person. Employees were organized into 8 divisions which were further divided into a total 21 functional workgroups, each led by a supervisor. Half of these workgroups (groups: *k* = 11; employees: *N* = 167) were randomly assigned to receive the SHIP intervention; the other half represented a control condition that received no intervention (groups: *k* = 10; employees: *N* = 125). Surveys and health assessments were administered prior to the intervention time period (baseline) and then again 12 months later (follow-up). Of the 292 employees in the organization, 264 (90%) participated in either the preintervention (*N* = 227) or postintervention (*N* = 198) data collection periods, and 167 participated in* both* baseline and follow-up.  Figure 1 provides a Consort diagram for the study.

Participants were predominantly male (90%) and white (79%; 2% Hispanic or Latino) with 97% having completed high school and 54% with college experience. The average age of participants was 45.13 years (SD = 9.60). Many participants were married (60%) or living with a significant other (12%); 55% indicated having children at home, and 33% indicated that they care for an adult relative. Participants had worked in their current job on average 11.4 years (SD = 8.5 years), and most (80%) reported working 40 hours per week. Participants' organizational roles were self-identified as supervisor (8.3%), crew leader (13.4%), crew member (70.5%), and others (5.1%).

### 6.2. Intervention Description

**Figure 2:**
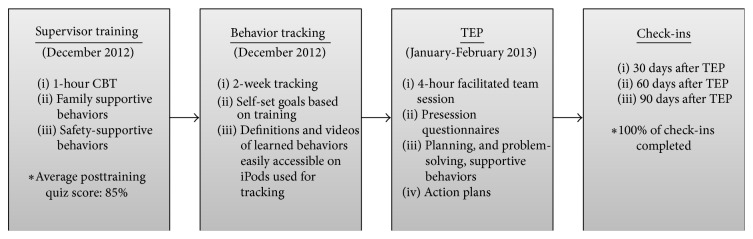
SHIP components. CBT: computer-based training, TEP: team effectiveness process.

The intervention examined here targets the entire work group, that is, both supervisors and workgroup members. First, we will describe the two components targeted at supervisors only. These included computer-based training focused on FSSB and SBS using the cTRAIN platform, followed by tracking of trained behaviors (completed December 2012). Second, we will describe the workgroup planning and problem solving team effectiveness process (TEP; completed January-February 2013) and the subsequent 30-, 60-, and 90-day post-TEP check-ins.  Figure 2 depicts these intervention phases.

#### 6.2.1. FSSB and SBS Computer-Based Supervisor Training

Team supervisors in the intervention condition first completed a 1-hour computer-based training program using the cTRAIN training platform. The cTRAIN platform was designed using proven behavioral training principles and is a self-paced, interactive training, with frequent quizzes and informative feedback [64]. Content was based on Hammer and colleagues [8] FSSB training and Zohar and Luria's [2] SBS training. Our preliminary research has demonstrated that the effectiveness of the FSSB training program to improve worker health using a computer-based training methodology [8]. The SBS training module integrates methods used in the only published safety intervention that has focused on improving safety climate by improving communication skills between supervisors and team members around safety issues [2]. Training included lessons on supervisor behaviors in the following areas: (1) emotional support, (2) daily job and personal problem solving, (3) family-supportive role modeling, (4) creative work-family management, (5) safety communication, (6) feedback/reinforcement and coaching, (7) providing resources, and (8) safety role modeling.

#### 6.2.2. Behavior Tracking

Next, supervisors chose specific training-related behaviors, based on the lessons noted above, that they wanted to improve. These behaviors were self-monitored and tracked for two weeks using HabiTrak tracking software, which had been preloaded onto an iPod Touch. HabiTrak technology was designed based on decades of research showing that transfer of knowledge is greater when individuals set goals and observe, evaluate, and record aspects of their own behavior (e.g., [18, 65–69]). The HabiTrak program guided supervisors through the behavior change process; users could access a detailed history of their behaviors and receive support through a help tab that provided behavioral definitions and video instructions for the specific learned behaviors. This program has been proven effective to transfer training in past research [18].

#### 6.2.3. Team Effectiveness Process (TEP)

We drew on the suggestions of Zohar and Luria [59] and Kelly and colleagues [9], to integrate work teams into the change process. Accordingly, we used a modification of an existing team intervention, the team effectiveness process (TEP; [70–72]) developed by Work Family Directions, a consulting firm that specializes in work-family integration practices within organizations. The TEP process involves an initial team assessment (brief paper-and-pencil survey of team practices, work-life effectiveness, and sources of overwork and inefficiency), followed by a four-hour team session led by a WFD-trained facilitator. These sessions apply social support and locus of control theory [73] to improve team planning and problem solving and to encourage supportive behaviors related to safety, health, and work-life balance within teams, including their supervisors. During the TEP session, team members utilized a variety of group problem solving methods including a review of assessment results, root cause analysis, brainstorming solutions, small group discussion, and voting on key issues. Conversations focused on designing new ways of performing essential tasks, identifying and eliminating low-value work and increasing focus on safety and positive work-family management behaviors. Each team developed an action plan outlining what steps they would take to make improvements, who was going to be responsible for those steps, and when those actions would be completed. Teams also developed operating principles summarizing team agreements regarding effectiveness, safety, and work-life balance supportive behaviors. Some examples from the teams include “We will have regular crew meetings on the job site,” “We will encourage questions from new workers,” and “We will respect each other's personal issues.”

#### 6.2.4. Check-Ins

Supervisors met with their teams 30, 60, and 90 days following the TEP session to review the team's operating principles, assess their progress, and update their action plans. The supervisor followed a Check-In Meeting Guide which included assessing change in morale and work attitudes, efficient use of time and resources, focus on safety practices, and communication within the team. Success stories, “win-wins,” and best practices were also noted.

### 6.3. Data Collection Procedure

Baseline data were collected October–December 2012 and the 12-month postintervention assessments were conducted October–December 2013. Data collection occurred at the work site during company time. Employees were informed that they were being invited to participate in a research study about factors affecting employees' safety, health, and work experiences. Participation was voluntary, and each employee received a $25 gift card from the researchers for completion at each data collection session. To match surveys across time points, employees were assigned unique identification codes based on an employee roster provided by the organization.

Assessments included a paper-and-pencil survey and objective health measures including blood pressure. Concurrent with survey completion, employees were called one at a time to complete the health assessments. A copy of all health measures was provided to the participants, as well as information for how to read and interpret the results. Protocols were in place for occurrences of high blood pressure (>160/90 mm Hg), and additional information including the phone number to a medical doctor was given to those participants.

## 7. Measures

### 7.1. Process Evaluation: Follow-Up Sessions at 30, 60, and 90 Days

All teams completed the 30-, 60-, and 90-day check-in meetings which were led by the supervisor with assistance from the trained facilitator(s). After each check-in meeting, the supervisor completed a form which included updates to their action plan and ratings of changes in the team on a 3-point scale from “No improvement” to “Great improvement.” These measures included morale and work climate, efficient use of time and resources, focus on safety practices, and communication within the team. Supervisors also made qualitative notes about success stories, “win-wins” and “best practices.”

### 7.2. Effects Evaluation: Reactions, Learning, Behavior, and Results

Measures of supervisor computer-based quiz results (learning), safety behaviors (behaviors), and perceived health (results), as well as onsite physical health assessments (results), were taken at baseline and twelve months after intervention from both intervention and control teams.

#### 7.2.1. Supervisor Computer-Based Quiz Scores

Learning was assessed as part of the computer-based training through embedded quiz questions at the end of each subsection. A final overall score was computed for each manager based on the posttest quiz score.

#### 7.2.2. Safety Behaviors

Safety compliance and safety participation behaviors were each measured with three-item, self-report scales [63]. A sample item from the safety compliance measure is “I use the correct safety procedures for carrying out my job.” A sample item from the safety participation measure is “I voluntarily carry out tasks or activities that help to improve workplace safety.” Responses to the items were on a 5-point scale with options ranging from 1 = “Strongly Disagree,” through 3 = “Neutral,” to 5 = “Strongly Agree.” Scale scores were computed as the mean numeric item response with higher scores indicating higher levels of safety compliance and participation. Scale scores for the safety compliance (coefficient-alpha = .92 at baseline and .91 at follow-up) and safety participation (coefficient-alpha = .86 at baseline and .89 at follow-up) measures demonstrated acceptable levels of measurement reliability at both assessment periods.

#### 7.2.3. Self-Reported Health

Physical health was measured by the physical health composite score from the SF-12 [74], a 12-item self-report inventory. A sample item is “In general, would you say your health is:” with item response options ranging from 1 = “Poor” to 5 = “Excellent.” The physical health composite score is a weighted composite of the 12-item responses with higher scores indicating higher levels of physical health; physical health composite scores are population normed to have a mean of 50 and a standard deviation of 10. Scale scores for the physical health composite (coefficient-alpha = .76 at baseline and .81 at follow-up) demonstrated acceptable levels of measurement reliability at both assessment periods.

#### 7.2.4. Blood Pressure

Blood pressure was measured using an Omron HEM-907EL machine, with an arm cuff. Three consecutive readings (with a one-minute rest in between) and an overall average were recorded. Mean blood pressure, defined as 1/3 systolic blood pressure + 2/3 diastolic blood pressure, was calculated for each participant's average reading. This measure of blood pressure has been shown to predict cardiovascular disease and death and may be the best predictor of these health outcomes when single blood pressure parameters are used [75]. In addition to these blood pressure measurements, participants were asked whether they were currently taking blood pressure medication. Use of such medication was used as a control variable in the analyses examining blood pressure as a dependent variable.

## 8. Results

Table 1 provides descriptive statistics for the various study variables at baseline and at the 12-month follow-up by intervention condition. No significant differences across intervention conditions were observed at baseline for blood pressure (*B* = 0.31, *p* = .84), SF-12 physical health composite scores (*B* = 1.23, *p* = .18), safety compliance (*B* = −0.08, *p* = .42), and safety participation (*B* = −0.29, *p* = .99). Given the lack of significant differences across groups at baseline, we next interpret some overall patterns in the data ignoring intervention group membership.

In addition to expected large correlations for the same variables over time, we observe, as might also be expected, negative correlations between age and SF-12 physical health composite scores at both time points, a negative correlation between taking blood pressure medication and SF-12 physical health composite scores at both time points, and a positive correlation between taking blood pressure medication and age. We also observe significantly higher mean blood pressure levels at baseline (*p* = .001) and 12 months (*p* = .01) than what would be considered normal (i.e., with a 120/80 mm Hg reading as “normal,” the mean blood pressure should be 93.2). Furthermore, mean SF-12 physical health composite scores were significantly lower at baseline (*p* < .001) and at 12 months (*p* = .002) than the population-normed mean value of 50. Thus, this sample appears to be less healthy than what is considered normative on these metrics.

### 8.1. Missing Data and Analytic Strategy

Of the 264 participants, 61 (33 intervention; 28 control) participated only at baseline, 36 (11 intervention; 25 control) participated only at the 12-month follow-up, and 167 (104 intervention; 63 control) participated at both baseline and follow-up (see Figure 1 for the Consort diagram). Thus, there is a notable amount of missing data. Several analyses were conducted to explore patterns in the missing data using demographic variables and the safety and health variables under investigation. Missing demographic variables assumed invariant over time (e.g., ethnicity and gender) and logically structured over time (e.g., age) were imputed based on the available variable value at the observed assessment wave. Those who participated only at baseline were on average significantly older (*M* = 48.60) than those who participated only at follow-up (*M* = 42.67) and at both assessments waves (*M* = 44.41), *F*(2,259) = 5.78, *p* = .004. Furthermore, those who participated at baseline only were more likely to take blood pressure medication at baseline (36.1%) than those who participated at both baseline and follow-up (22.3%), *χ* ^2^(1) = 4.40, *p* = .036. No other variables varied significantly across these three participant groups (i.e., those who participated only at baseline, those who participated only at follow-up, and those who participated at both time points).

In light of the amount of missing data and the noted patterns across participation groups, we used the full-information maximum likelihood routine available in Mplus 4.2 to estimate intervention effects. The advantage of this missing data approach over typical software default options, such as listwise deletion, is that the full-information approach provides more appropriate parameter estimates and standard errors when the data are missing-at-random (MAR). Although there are no available tests for the MAR assumption, the plausibility of this assumption increases if observed variables related to both the likelihood of missingness and the values of other observed variables without missing data are used in the analytical model [76]. Thus, we include as control variables in each model the value of the safety and health outcome variable at baseline as well as participant age; for the models testing for intervention effects on blood pressure and physical health, we also include an indicator of blood pressure medication use at baseline.

Finally, the lack of independence due to the nesting of employees within divisions or workgroups was assessed. We used the intraclass correlation (ICC) to quantify the degree of nonindependence for the four safety and health outcomes. For all four outcomes, Mplus estimated ICCs near zero (max ICC = .003). Thus, in the presented models, we do not estimate division- or workgroup-level random effects; the parameter estimates from the models that included these random effects were substantively identical to those reported.

### 8.2. Evaluation of Intervention Implementation

All supervisors in the intervention condition completed the computer-based training. To do so, supervisors needed to answer correctly each of the periodic quizzes in the training to continue to the next training topic; incorrect responses required the supervisor to repeat that training section before continuing. The average score on the final training knowledge test was 85% indicating an adequate knowledge training outcome [15]. All supervisors in the intervention condition reported using the behavioral self-monitoring tools. Although this data was not collected from the supervisors, poststudy interviews yielded some insights on the ease and difficulty of this task. Supervisors found it easier to provide emotional support (e.g., taking time to talk to employees) and role modeling (e.g., leaving work on time and avoiding coming into work on weekends); supervisors found it more difficult to provide resources to manage conflicts (e.g., due to budget and staffing constraints).

All workgroups assigned to the intervention condition completed the TEP sessions. Furthermore, all workgroups successfully completed the 30-, 60-, and 90-day check-in tasks following these TEP sessions. At 60-day check-ins, 90% of teams reported some or great improvement to morale and work attitudes; 70% of teams reported some improvement in more efficient use of time and resources; 100% of teams reported some or great improvement to increased focus on safety practices within the team; and 100% reported some or great improvement to communication within the team. Examples of topics that these workgroups identified and worked on include institute end of day jobs review meeting, implementing new job priority system, institute “ride alongs” for Traffic Control to educate them to safety risks, and conducting preconstruction meetings on specific projects to avoid emergencies and inefficiencies. When asked about success stories, supervisors made comments such as “More communication between crew members,” “One staff member wearing safety vest more,” and “Sharing lining process with other sections gets them what they need and removes misunderstanding for crews.”

### 8.3. Tests of Intervention Effects

Table 2 provides the results of the analysis of intervention effects on the safety and health outcomes. For the safety outcomes, no significant intervention effects were observed. Despite trending in the expected direction, mean safety participation scores at the 12-month postintervention assessment period were not significantly higher in the intervention workgroups than in the control workgroups after controlling for baseline safety participation scores and age (*B* = 0.14, *p* = .12, Δ*R* ^2^ = .014). Similarly, mean safety compliance scores at the 12-month postintervention assessment period did not differ significantly among the intervention and control workgroups controlling for baseline safety compliance scores and age (*B* = −0.02, *p* = .83, Δ*R* ^2^ = .001). Thus, hypothesis 1 was not supported.

For the health outcomes, mean blood pressure scores at the 12-month postintervention assessment period were significantly lower in the intervention workgroups than in the control workgroups controlling for baseline blood pressure scores, age, and use of blood pressure medication (*B* = −2.15, *p* = .038, Δ*R* ^2^ = .015). In contrast, mean SF-12 physical health composite scores at the 12-month postintervention assessment period did not differ significantly among the intervention and control workgroups controlling for baseline SF-12 scores, age, and use of blood pressure medication (*B* = −0.32, *p* = .69, Δ*R* ^2^ < .001). Thus, hypothesis 2 was partly supported.

## 9. Discussion

The present study found evidence of SHIP intervention effects on one of the two health indicators (i.e., blood pressure) and neither of the two safety indicators. Descriptively, the size of the intervention effect on blood pressure—considering the value of the Δ*R* ^2^ statistic and a difference of 2.15 mmHg—would be considered small in size. While this decrease is in the lower end of the range of systolic BP losses found in a meta-analysis of the impact of weight reduction on blood pressure [77], it is well above the lowest. Furthermore, in the health domain, small effects can be important. Indeed clinical trials have been stopped on ethical grounds with even smaller effect sizes (e.g., [78–80]). Thus, we consider this effect as important because of the profound effects of elevated blood pressure over hundreds of workers.

SHIP was an intervention designed to reduce psychosocial risk factors of work-life stress and poor safety communication. While the specific risk factors were not assessed as outcomes, we can conclude that SHIP was successful in improving blood pressure, a health outcome more closely aligned theoretically with a reduction in work-life stress rather than with an improvement in safety communication. Therefore, we believe that there is evidence to suggest that the SHIP components of FSSB, SBS, and TEP, together, led to improvements in blood pressure over time. Future research should examine ways of improving SHIP to more directly target safety outcomes and to examine in more detail the processes, both psychological and physical, through which SHIP operates.

A limitation of the integrated intervention examined in the present study is that the two intervention components (supervisor training and TEP), while reinforcing one another, cannot be teased apart as to whether the intervention effects are due to the supervisor training component, the team-based component, or both. In the design of this study, we erred on the side of creating an intervention that would be successful on the belief that programs in organizations are most successful that involve top-down (i.e., supervisor-based) and bottom-up (i.e., employee-based) components.

Although the apparent lack of intervention effects on self-reported safety behaviors is disappointing, inspection of the baseline mean scores for these variables shown in Table 1 suggests one possible explanation for the lack of safety-specific intervention effects, namely, a ceiling effect. Indeed, the mean baseline score for safety compliance (*M*_baseline_ = 4.14) is close to its maximum possible value of 5. The mean baseline score for safety participation (*M*_baseline_ = 3.64, again with a maximum of 5) is not as high as that for safety compliance but is still well above the theoretical scale midpoint. Both indicate general agreement that participants on average comply with and participate in safety-related considerations. Thus, there was not much room for potential improvement, at least as measured through these instruments. These baseline means likely reflect some awareness of the importance of safe work routines and the effects of prior safety training, critical issues in an industry with a high injury risk. Additionally, based on our qualitative data from the 60-day check-ins, 100% of teams reported some or great improvement to increased focus on safety practices within the team; and 100% reported some or great improvement to communication within the team.

We view the nonsignificant but trending intervention effect on safety participation, where there is likely less of a ceiling effect due to the somewhat lower baseline mean, as promising and worth exploring in future research with a larger sample. Indeed, safety participation may be a key factor in maintaining a safe workplace because it suggests a concern for the safety of coworkers and the team. Further, it may be that the nature of the intervention, focused on changing the safety climate through supervisors, could have changed the standard participants used to assess their safety behaviors. Such “beta change” among participants can actually make the detection of change more difficult even with successful interventions [81].

In sum, we suggest that this study offers a first look at an intervention that integrates both safety and work-life balance supervisor training, along with a team-based approach that is focused on team effectiveness process for planning and problem solving in an effort at reducing psychosocial risk factors and in turn improving safety and health of workers. We believe that while this is a first step, it is an important contribution to the literature because there are few workplace interventions aimed at the reduction of psychosocial risk factors that have been evaluated using scientifically sound research designs. These findings add to the existing work-life stress reduction intervention research on the effectiveness of FSSB training [8, 9] and extend this work to the examination of team-based methods using the TEP approach, as well as integrating safety communication training drawing on methods used by Zohar and colleagues [2, 10, 59–61]. We suggest that while the findings for safety were not significant, this could be due to particularly strong safety behaviors in place at baseline leading to possible ceiling effects. Thus, we would not abandon the safety communication training portion of SHIP but rather suggest that future research is needed.

We believe that to effectively develop workplace interventions that lead to improved health and safety of workers, we need to replicate, customize for different industries, and better understand the processes that are at play. This is just the start of a research program on the effectiveness of SHIP. Questions related to strength of intervention, psychological processes, and behaviors impacted are all part of our future research program as we currently work towards extending this research to additional industries and populations. As stated by Biron et al. [82] “… information on how to develop effective strategies to reduce or eliminate psychosocial risks in the workplace is much more scarce, ambiguous and inconclusive.” We believe that the findings from this research suggest that an intervention focused on supervisor support training and a team effectiveness process for planning and problem solving can improve a critical area of employee health and this positive effect suggests that a continued strengthening and targeting of the intervention could expand the impact to further improve employee health and safety.

In the end, we believe that our intervention offers important insight into ways that the psychosocial workplace risk factors, at least that are associated with work-life stress, can be impacted leading to improved health of workers as demonstrated by significant reductions in blood pressure. This type of preventative approach, combined with more awareness around health promotion activities and behaviors, is what NIOSH is working to advance with the focus on TWH.

## Figures and Tables

**Table 1:** Estimated means, standard deviations, and correlations among study variables by intervention condition.

Variable			(1)	(2)	(3)	(4)	(5)	(6)	(7)	(8)	(9)	(10)
	M	M	47.51	48.10	95.78	94.41	3.63	3.75	4.12	4.11	44.87	0.25
		SD	6.67	7.07	11.97	10.18	0.82	0.81	0.67	0.56	9.11	0.44
(1) Health (baseline)	46.52	6.68	(.76)	.61^*∗*^	−.16^*∗*^	−.21^*∗*^	.08	.08	.08	−.05	−.23^*∗*^	−.31^*∗*^
(2) Health (12 m)	48.14	6.73	.53^*∗*^	(.81)	−.31^*∗*^	−.30^*∗*^	−.03	.08	−.06	.11	−.23^*∗*^	−.35^*∗*^
(3) Blood pressure (baseline)	95.45	9.86	−.05	.18	1.00	.83^*∗*^	−.03	.07	.01	−.09	.14	.09
(4) Blood pressure (12 m)	95.81	9.89	−.06	−.15	.58^*∗*^	1.00	−.15	−.07	−.06	−.08	.09	.10
(5) Safety participation (baseline)	3.65	0.81	.02	.05	.02	.13	(.86)	.60^*∗*^	.53^*∗*^	.40^*∗*^	.15	.05
(6) Safety participation (12 m)	3.67	0.84	−.18	−.03	.07	.11	.82^*∗*^	(.89)	.37^*∗*^	.64^*∗*^	−.04	−.13
(7) Safety compliance (baseline)	4.19	0.62	.09	−.10	.05	.25^*∗*^	.51^*∗*^	.42^*∗*^	(.92)	.49^*∗*^	−.05	.15
(8) Safety compliance (12 m)	4.17	0.70	.14	.11	.00	.07	.52^*∗*^	.62^*∗*^	.62^*∗*^	(.91)	−.05	−.02
(9) Age in Years	45.50	10.13	−.14	−.27^*∗*^	.10	.03	.13	.13	.14	.06	1.00	.38^*∗*^
(10) Blood pressure medication	0.27	0.45	−.29^*∗*^	−.42^*∗*^	−.06	−.10	−.08	.07	.01	.21^*∗*^	.31^*∗*^	1.00

Notes: ^*∗*^ *p* < .05. Intervention *N* = 148; Control *N* = 116. Intervention condition information above main diagonal; Control condition information below main diagonal; Blood Pressure Medication (Yes = 1, No = 0). Estimates are based on full-information maximum likelihood estimation to account for missing data values. Diagonal entries in parentheses are Cronbach's alpha reliability coefficients.

**Table 2:** Model results for intervention effects on safety and health outcomes.

Predictor	12-month safety outcomes		12-month health outcomes	
	DV: safety participation	DV: safety compliance	DV: blood pressure	DV: physical health
	Coefficient (SE)	Coefficient (SE)	Coefficient (SE)	Coefficient (SE)
Intercept	1.37^*∗*^ (0.28)	2.13^*∗*^ (0.31)	32.87^*∗*^ (4.95)	24.03^*∗*^ (3.20)
Age	−0.01 (0.01)	−0.00 (0.01)	−0.03 (0.06)	−0.06 (0.05)
Blood pressure medication	·	·	0.29 (1.33)	−3.44^*∗*^ (1.09)
DV at baseline	0.69^*∗*^ (0.05)	0.51^*∗*^ (0.06)	0.68^*∗*^ (0.05)	0.55^*∗*^ (0.07)
Intervention	0.14 (0.09)	−0.02 (0.08)	−2.15^*∗*^ (1.03)	−0.32 (0.82)

Residual variance	0.35^*∗*^ (0.04)	0.27^*∗*^ (0.03)	45.98^*∗*^ (4.95)	28.93^*∗*^ (3.20)
Model *R* ^2^	.49^*∗*^	.30^*∗*^	.55^*∗*^	.42^*∗*^

Notes: ^*∗*^ *p* < .05. *N* = 264. Intervention (intervention = 1, control = 0); blood pressure medication (yes = 1, no = 0). Models use full-information maximum likelihood routines to estimate parameters accounting for missing data.
